# Research on left hard shoulder width of super multilane highway based on PTSU operation

**DOI:** 10.1371/journal.pone.0287606

**Published:** 2023-06-23

**Authors:** Zifan Ni, Jianxiao Ma, Tao Lu, Penghui Zhao

**Affiliations:** Nanjing Forestry University, Nanjing, China; Southwest Jiaotong University, CHINA

## Abstract

Part-time shoulder use (PTSU) is a traffic strategy that temporarily uses the shoulder as a lane when necessary. Research has shown that, when a hard shoulder is required to set the traffic function, the left hard shoulder is preferable. Super multilane highways are usually equipped with left hard shoulders of sufficient width, but the wide cross-sectional characteristics make it difficult for vehicles to turn into the emergency parking lane to avoid a breakdown or accident in the lane, which is an ideal implementation object of PTSU. In this study, two virtual simulation scenarios for PTSU were created: one with the left hard shoulder open and used as a travel lane, and the other with the left hard shoulder closed and its original function restored. Vehicle driving data were collected through driving simulation experiments to reveal the influence of the left hard shoulder on vehicle handling stability. The optimal width of the left hard shoulder was determined by ANOVA and comparison of the mean and standard deviation. The purpose of this study was to quantify the effect of the width of the left hard shoulder on the driving stability of vehicles in the inside lane under PTSU and determine the ideal shoulder width by comparing the stability parameters of vehicles.

## Introduction

To alleviate traffic problems, such as highway congestion, secondary accidents caused by congestion, accident black spots, and bottleneck points caused by geometric alignment, several countries have implemented part-time shoulder use (PTSU) on high-volume highways to maximize the effectiveness and efficiency of facilities or road networks while improving traffic throughput and safety. PTSU is a transportation system management and operation strategy that permits the left or right shoulders to be used as travel lanes during specific hours of the day [1]. The Federal Highway Administration (FHWA) categorizes PTSU operation into three modes: BOS (available only to authorized buses), S-PTSU (available only to vehicles at scheduled times), and D-PTSU (dynamic opening of road shoulders based on real-time traffic conditions). This study focuses on S-PTSU and D-PTSU. In the S-PTSU and D-PTSU modes, the shoulder is only open for use during times of the day when the highway is likely to be heavily congested (such as peak hours), when congestion is detected, or when general-purpose lanes are closed because of construction activities or accidents. When the shoulder is no longer required as an additional carriageway, it will be closed and restored to its original function, including its function as a shoulder and as an emergency lane to provide emergency parking space for vehicles while retaining and recognizing the shoulder’s basic physical characteristics.

The cross-sectional flow of a super multilane highway is huge, and the speed of the inner lane is high (above 100 km/h). When an accident occurs in the inner lane, the vehicle involved may lose control and come to a halt in the inner lane, posing a risk of secondary accidents. PTSU can assist in the use of early warning and induction facilities, as well as guide the vehicles in the inner lane upstream of the early warning safety area in the accident section to drive into the left hard shoulder in advance to avoid dangerous regions. The traffic capacity of the original road is restored to a certain extent, while safely traveling through the accident-affected region. The Shoulder Geometrics and Use Guidelines (NCHRP Report 254) investigated the shoulder utilization rate of a bidirectional 8-lane segment of Interstate 95 between I-495 and I-695. It was discovered that only approximately 15% of vehicles parked in the nonservice sector of the road use the left hard shoulder, indicating that the safety space provided by the left hard shoulder has some demand but is significantly lower than that of the right hard shoulder [2]. When the hard shoulder is required to set the traffic function, the right hard shoulder should be retained preferentially from the emergency parking function unless there is sufficient width outside the right side of the hard shoulder to provide additional shoulders. If there is enough space, the left hard shoulder is usually converted into a full lane when additional lanes are required. The advantage of using the left hard shoulder with PTSU is that it can avoid the interference of most large vehicles. This is because on multilane highways, large vehicles are frequently limited to several lanes on the right side of the highway, whereas the left hard shoulder is not easily affected by the ramp unless there is an entrance or exit on the left side of the highway. However, the width of the left hard shoulder is frequently less than that of the right shoulder and providing a width of more than 12 ft (3.66 m) is very difficult.

The “Policy on Geometric Design of Highways and Streets” [3] provides a guideline design specification for the width of the left hard shoulder, which shall not be less than 10 ft (3.05 m) for multilane highways with six or greater number of lanes. When the average daily truck traffic volume exceeds 250 vehicles, the left hard shoulder width should be 12 ft (3.66 m). In addition, the design of the left hard shoulder in China is mainly based on the “Technical Standard of Highway Engineering” (JTG B01-2014) [4], which stipulates that the width of the left hard shoulder should not be less than 2.5 m for highways with eight or greater number of lanes. However, most studies have shown that numerous factors must be considered when setting the width of the left hard shoulder. The number of lanes is an influencing factor that needs to be explored when setting a hard left shoulder on a freeway. An increase in the number of lanes on a freeway can lead to an increase in the total number of crashes [5]. The change in the width of the left hard shoulder also has a significant impact on the accident rate [6]; therefore, the width of the left hard shoulder cannot simply rely on a quantitative specification, but needs to be decided according to the actual situation of the highway. Early studies on the width of the left side hard shoulder were mainly based on a comparative analysis of the number of accidents on highways with different shoulder widths set up to obtain the influence law of shoulder width on the change in accident rate. However, owing to their early research time, the highways involved in the studies were mostly ordinary highways with four or smaller number of lanes in both directions. In recent years, research on the hard shoulder on the left side of multilane freeways has mainly focused on investigating the driving characteristics of vehicles and physiological characteristics of drivers. Zhao et al. [7] collected data on the eye movement and heart rate of subjects as well as the vehicle lateral position through driving simulation experiments. They evaluated the safety impact of the hard shoulder on the left side through data analysis. The results showed that the width of the hard shoulder on the left side has a significant impact on vehicle operation, and the ideal width of the left hard shoulder of a two-way eight-lane highway should be 0.75–1.5 m. However, such studies were focused on narrow shoulders in which PTSU is not applicable owing to the width limitation. Regarding the research on shoulder width for PTSU, Jenior et al. [1] proposed an adjustment factor for the width of the left hard shoulder with PTSU operation based on the type and severity of the accident, and the resulting adjustment factor is applicable to shoulder widths in the range of 0.7–11.0 ft. When the shoulder width exceeds 12 ft, its adjustment factor does not change again. However, the adjustment factor is only applicable to freeways with number of through lanes ranging from two to seven, and it is not applicable to super multilane freeways with eight or greater number of lanes.

Driving simulation experiments can eliminate environmental disturbances and effectively evaluate road safety [8]. Rosey et al. [9] proved that the width variation of the shoulder has a significant effect on driving parameters such as the lateral offset and speed of vehicle. With the application and implementation of PTSU, the optimal width of the left hard shoulder under different conditions requires comprehensive analysis and guidance. Therefore, this study considered a ten-lane highway as an example, and driving simulation experiments were conducted to investigate the ideal width of the left hard shoulder under different conditions. By analyzing the effect of the width for two different left hard shoulder scenarios on vehicle driving stability, the optimal width of the left hard shoulder of a two-way ten-lane highway with PTSU was established with the aim of improving the flexibility of using the left hard shoulder and safety of super multilane highways implementing PTSU.

## Literature review

### Left hard shoulder width

The majority of early studies on the left hard shoulder were based on historical data of traffic accidents on real roads and traffic environments, and the impact of the hard shoulder setting methods on accident rates was studied using comparative analysis. Most of the studies focused on the right shoulder, and only few studies tackled the left shoulder. Hadi et al. [10], Noland and Oh [11], and Thomson et al. [12] studied the effect of increasing or decreasing the shoulder width on the change in accident rate. The results indicated that widening the left hard shoulder can reduce the accident rate. Dewar and Olson [13] explained the reasons for this by comparing the crash rates on 37 two-lane roads in California with shoulder widths of 2, 4, and 8 ft. They showed that narrow shoulder widths on both sides cause drivers to move away from the shoulder and closer to the center of the road, leading to an increased likelihood of collisions with vehicles in other lanes. However, the opposite conclusion was obtained by Urbanik and Bonilla [14], who reported that the reduction in the left hard shoulder width from 8–12 ft (2.44–3.66 m) to 1–3 ft (0.31–0.91 m) reduced the crash rate for most of the roadways. This might have been because of the effective width of the left hard shoulder. Ksaibati and Crowe [15] conducted a 5-year study in Wyoming on the effects of hard shoulders on both sides of the road on highway safety. They concluded that a hard shoulder width of 0.6 m (2 ft) from the edge of the travel lane was the most effective in reducing the accident rate (19% reduction in the accident rate). The possible reason for this is the influence of the width of the adjacent lane. Dixon et al. [16] reported that the width of the left hard shoulder was important when the adjacent lane was 11 ft (3.35 m) wide but not when it was 12 ft (3.66 m) wide. A left hard shoulder width above a certain value also causes a significant increase in the crash rate. Bamzai et al. [17] used the Illinois highway crash data from 2000 to 2006 to establish a relationship between crashes occurring on the shoulder and the width of the hard shoulder on both sides. They concluded that increasing the width of paved shoulders from 4 to 6 ft could reduce fatal and injury crashes on urban multilane highways by 7–17% and 0–2%, respectively.

### Study of vehicle and driver parameters based on driving simulation experiments

Although the conventional research method of comparing traffic accident data can help determine the effectiveness of the left hard shoulder, it is not possible to compare the method of setting the left hard shoulder, which has some limitations, under exactly the same conditions. Driving simulation technology can eliminate the negative impact of confounding factors on experimental data and is an effective tool that has been widely used in recent years for studying the width of the left hard shoulder. Liu et al. [18] tested the effect of the lane and shoulder width on the driving behavior for a three-lane urban underground expressway using a driving simulator. The results showed that the shoulder width had a significant effect on the driving speed and lateral offset, and narrower shoulder widths led to reduced vehicle speed and lateral offset. Ding et al. [19] investigated the effect of the inside shoulder width on vehicle operation based on driving simulation experiments. The analysis indicated that the inside shoulder width had no significant effect on the driver’s speed choice but had a statistically significant effect on the vehicle’s lane position, and that the vehicle’s lane position was negatively correlated with the inside shoulder width. They concluded that a 2.5 m wide medial shoulder allowed vehicles to stay in the center of the lane. Ben-Bassat and Shinar [20] evaluated the impact of the shoulder width and guardrail position on the driving stability using a driving simulator by selecting the actual speed, lane position, and perceived safe driving speed as evaluation indicators. The results showed that a narrow shoulder width can reduce the driving speed and is beneficial for maintaining a stable lane position; however, it increases the rate of accidents. Bella [21] and Van Der Horst and De Ridder [22] studied the effect of the left hard shoulder width on the vehicle speed and lateral position through driving simulation experiments. They found that the left hard shoulder width had a significant effect on both the vehicle speed and lateral position.

### Positive and negative benefits of PTSU

Studies on the effects of PTSU have mainly focused on the safety of highways, which is an important evaluation index; therefore, numerous studies related to highway safety have been conducted. Yang et al. [23] used the freeway traffic crash data obtained from Washington Department of Transportation to construct a multidimensional multilevel system for traffic crash analysis. Considering load balancing, the FP-growth algorithm was optimized in parallel using the Hadoop platform to achieve an efficient and accurate association rule mining calculation for massive amounts of traffic crash data. The causes of freeway traffic crashes were then identified and revealed based on the results of the coupling mechanism among the crash precursors. In addition, Yang et al. [24] built a dataset with six dimensions based on accident data and proposed the weighted orientated multiple dimension interactive Apriori algorithm. This improved algorithm was adopted to mine the association rules from the perspective of multidimensional interaction: full mapping crash cause and crash dimension autocorrelation perspectives to reveal the internal coupling mechanisms and differences between freeway traffic crashes in various area types. They introduced the random forest algorithm to identify the traffic flow variables of crash precursors and applied a support vector machine to build a traffic crash risk prediction model under the condition of temporal difference [25]. This technique aimed to better realize the change from static analysis after a crash to dynamic analysis before a crash toward freeway safety, as well as explore the relationship between dynamic traffic flow characteristics and real-time traffic crash risk under different temporal conditions.

Most studies have demonstrated that PTSU can bring positive benefits to roadways, although it also has some negative effects. Compared to physically enlarging highway sections, PTSU can efficiently boost road capacity while lowering expenses and right-of-way requirements [26]. PTSU can drastically reduce vehicle travel time on highways and offer a steadier traffic density across highway networks [27]. However, PTSU implementation increases the risk of collision in a variety of hazardous scenarios, leading to an increase in the accident rate [28]. Furthermore, the abrupt decrease in shoulder usage near the end of the PTSU route reduces the capacity of the downstream road by one lane, resulting in traffic congestion and considerable loss in speed [29]. Jenior et al. [30] summarized the performance evaluation reports from various types of highways in Europe and the United States following the implementation of PSTU. They reported significant improvements in capacity and traffic throughput during peak periods, as well as reductions in overall crash frequency and travel time on the highway; however, there was a slight increase in accidents in the PTSU segment. Guerrieri and Mauro [31] found that the activation of a hard shoulder running system did not lead to significant variations in general safety conditions despite its considerable benefits for highway functionality. Coffey and Park [32] reported that while PTSU improves road operation, it must be safe and structurally sound. PTSU is a strategy that can reduce congestion, car emissions, and paved lane use requirements through improved traffic flow. Choi et al. [33] discovered a positive relationship between the length of the hard shoulder running and changes in crash frequencies. In addition, the number of freeway travel lanes affects crash frequency at sites with PTSU. Samoili et al. [34] achieved a 10.6% capacity increase and maintenance of speed at 100 km/h by implementing PTSU.

Most previous studies did not focus on super multilane highways; thus, the width range of the left hard shoulder was less than 1.5 m, and widths equal to or greater than 2.5 m were rarely considered. Therefore, this study investigated the optimal value of the left hard shoulder width under the PTSU strategy by analyzing the driving stability of the inner lane of a two-way ten-lane highway with a left hard shoulder width ≥ 2.5 m.

## Methods

### Participants

A total of 160 volunteers were recruited for this study. To eliminate the impact of gender on the driving habit, all volunteers were male, who were not informed of the purpose of the experiment. The participants completed a form containing their personal information, including their age, years of driving experience, and average annual mileage on the highway. Approximately 70% of the participants aged over 24 years and under 50 years (average age = 35.025 years, standard deviation = 6.178 years) had at least 2 years of driving experience (average driving experience = 8.981 years, standard deviation = 4.163 years) with C1 license. Prior to the beginning of the experiment, all participants underwent a 15-minute driving simulator training to familiarize themselves with the environment and use of the simulator and to ensure that they would not experience any discomfort during the experiment. Written informed consent, as presented in S1 Appendix, was obtained from all participants before the experiment. The experiment was reviewed by the Ethics Committee of Nanjing Forestry University.

### Apparatus

**Fig 1:**
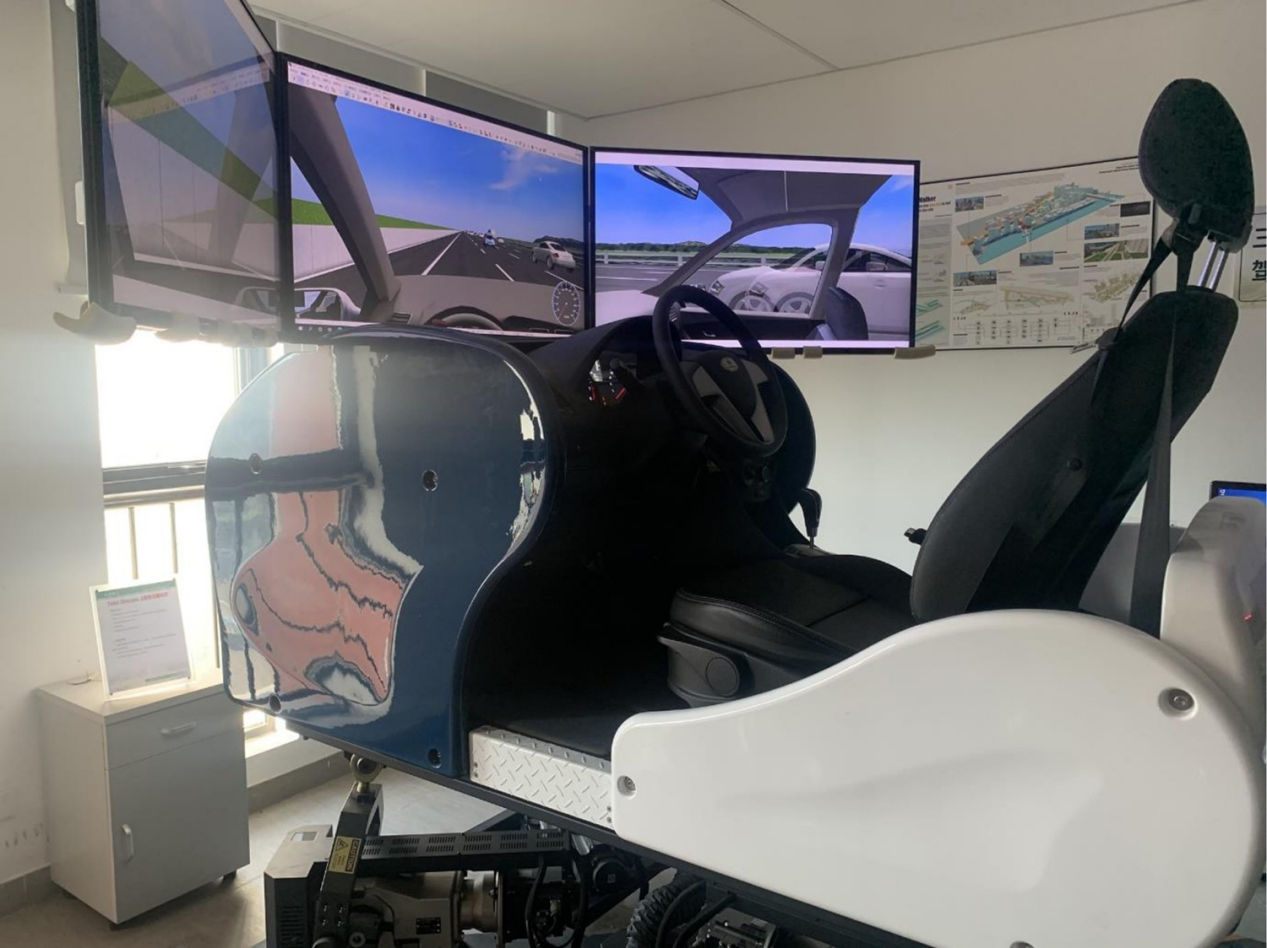
Driving simulator.

To eliminate the negative effects of confounding factors on the experimental data and ensure driver safety, the driving safety of the vehicle was analyzed through a driving simulation experiment. The driving simulator (Fig 1) used in this study was built by INNO Simulation. With the UC-win road software on board, the driving simulator was able to record vehicle operating data (including speed, acceleration, lateral offset, and transverse angular velocity) and driving behavior (gas pedal, brake pedal, steering wheel, etc.) at a frequency of 1–50 Hz, with the acquisition frequency set as 10 Hz. Different road scenarios were simulated using the driving simulator. During the experiment, virtual scenes were projected onto three large screens in front of the vehicle. These screens provided a field of view of approximately 140° in the front.

### Scenarios

**Fig 2:**
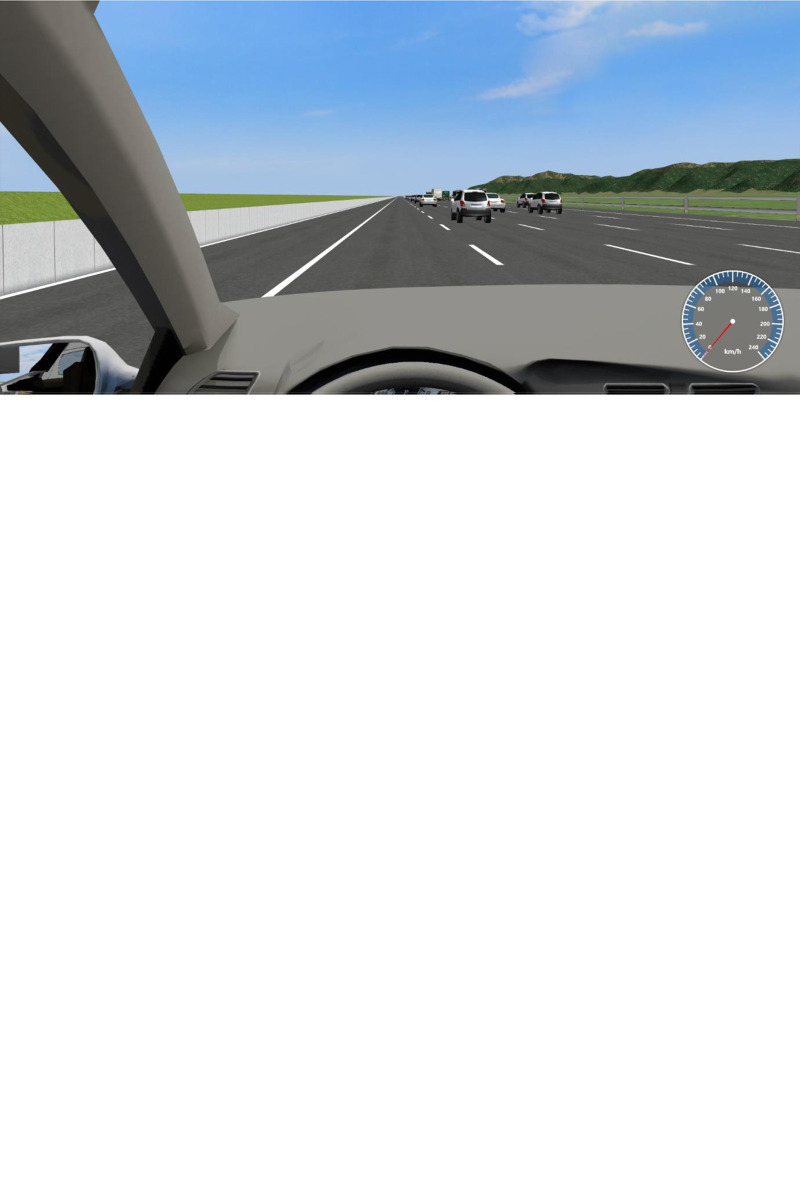
Three highway scenarios with different left hard shoulder widths. (a). 2.5 m width, (b). 3 m width, (c). 3.5 m width.

Whether the left hard shoulder is closed or open, its width affects the driving stability of vehicles in the innermost lane. To determine the impact of the left hard shoulder width in both states, two different scenarios were set up for the experiments to compare and establish the optimal width of the left hard shoulder. Three independent two-way ten-lane highways were set up in both scenarios, with the same geometric parameters except for the width of the left hard shoulder. According to the Technical Standards for Highway Engineering (JTG B01-2014), a left hard shoulder should be set on a two-way eight-lane highway and above, and the width of the left hard shoulder should not be less than 2.5 m. Therefore, the left hard shoulder widths of the three highway scenarios in this study were set as 2.5, 3, and 3.5 m. The three experimental scenarios constructed based on the width of the left hard shoulder are illustrated in Fig 2.

**Fig 3:**
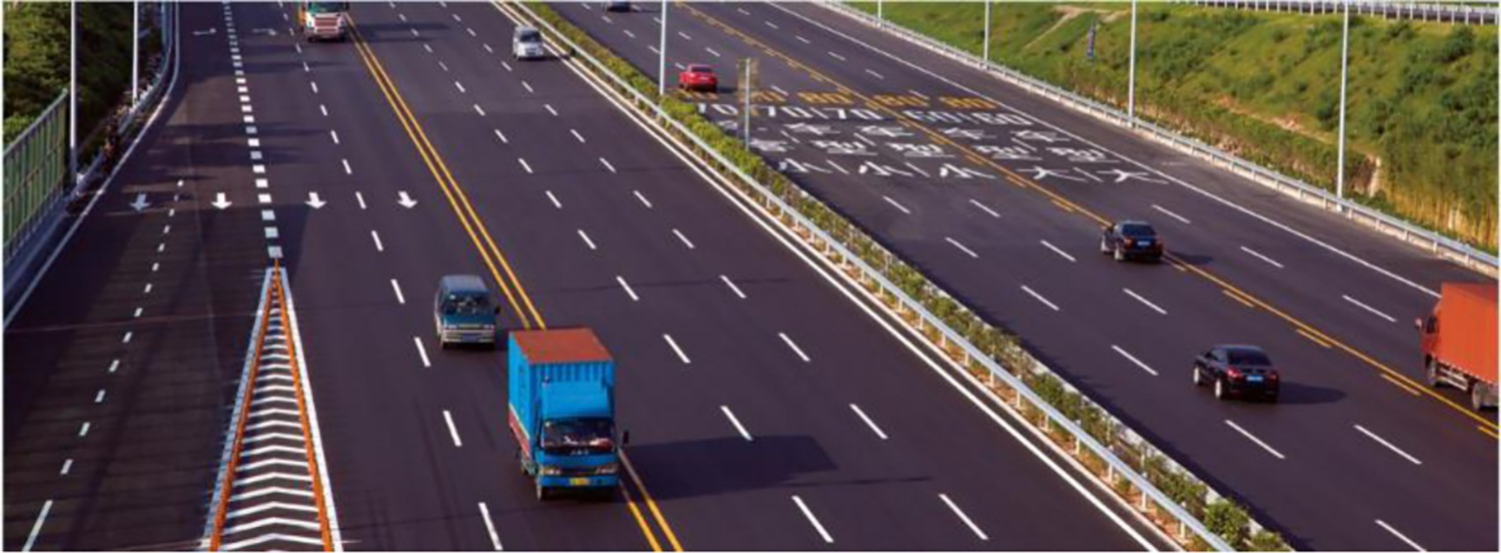
Shuijing–Guanjingtou Expressway.

The geometric parameters of the highway in the simulation scenario were based on the super multilane section of the Shuijing–Guanjingtou Expressway in Shenzhen, China (Fig 3), which has 10 lanes in both directions, a design speed of 100 km/h, and no left hard shoulder of sufficient width (there is insufficient width space for vehicles to stop or drive), and the highway parameters are listed in Table 1. The roads in the scenario were supplemented with three different widths of the left hard shoulders in addition to the original ones to determine the influence of various left hard shoulder widths on the driving stability. The lengths of all roads in the scenario were set to 6 km, and each road was independent, with the longitudinal section characteristics set to be the same so that the driving experience would not be affected by the terrain height. The geometric design parameters of the road scenarios are listed in Table 1. The virtual model of the car driven by the participants in the scenario was designed based on the size of a passenger car because the inner lanes of super multilane highways are often open only to passenger cars. When driving on one of the roads, the application would automatically stop and output data from the entire driving procedure.

**Table 1:** Geometric design parameters of the section.

Roadway parameters	
Number of lanes	10
Design speed	100 km/h
Design capacity	150,000 vehicles per day (minibuses)
Length	6 km
Lane width	3.75 m
Right shoulder width	3 m
Left shoulder width	2.5 m, 3 m, 3.5 m
Central divider width	2.5 m
Marginal strip width	0.5 m
Rate of cross slope	1.5%
Rate of longitudinal slope	2.5%

In the first scenario, the left hard shoulder of the highway was open and used as a temporary travel lane. To simulate a realistic traffic scenario while avoiding impact on the test vehicles, all lanes in the scenario except the lane where the test vehicles were located were set up with quantitative traffic flow based on the design traffic volume of a two-way ten-lane highway. In addition, a specific amount of traffic volume was set on the left hard shoulder, but only different types of passenger cars were set to simulate vehicle restrictions.

In the second scenario, the left hard shoulder was closed, its original function was restored, and no traffic volume was set. However, parking vehicles were set on the left hard shoulder at random intervals to achieve the simulation effect when the left hard shoulder was used as an emergency parking lane, and the traffic volume of the other lanes was set unchanged. In the two scenarios, large and heavy vehicles, such as trucks and large buses, were set in the outermost two lanes of the highway, and only various small and medium vehicles were set in the inner three lanes of the highway to simulate the traffic distribution on a real highway. Vehicles on the left hard shoulder only include small and medium sizes, as large vehicles traveling in the outside lane typically use only the right shoulder for emergency stops. Previous studies have shown that using the PTSU strategy to convert shoulders to lanes to increase the number of lanes may increase the accident frequency. The use of added lanes as HOV (High-Occupancy Vehicle) lanes, which can cause differences in speed between adjacent lanes, may be one explanation for the increase in accidents [5]. Therefore, the left hard shoulder is only open to small and medium vehicles to prevent large, slow-moving vehicles from unnecessarily affecting high-speed traffic in the inner lane.

Because large cars are restricted to the outer two lanes of the highway and small cars are concentrated in the inner three lanes, the traffic volumes in the second and third lanes (in left-to-right order) in the scenario are greater than those in the fourth and fifth lanes (no traffic flow was set in the innermost test lane). The traffic volumes in the second and third lanes were set to 1800 VPH, while those in the fourth and fifth lanes were set to 1300 VPH. The specific capacity classifications of PTSU are listed in FHWA’s simulated PTSU lane capacities (Table 2).

**Table 2:** FHWA’s simulated PTSU shoulder capacity.

Shoulder type	Shoulder capacity (VPH)
Short and low quality	1262
Long and low quality	1334
Short and high quality	1610
Long and high quality	1687

Short shoulders are defined as dynamic shoulders 305 m (1000 ft) long and below, including the length of the bottleneck section. Long shoulders are dynamic shoulders 2.4 km (1.5 miles) in length and above. Low-quality shoulders are 3 m (10 ft) wide and high-quality shoulders are 3.7 m (12 feet) wide. The traffic volumes set in the open left hard shoulder scenario are listed in Table 3. The traffic volumes in the other lanes are consistent with the closed left hard shoulder scenario.

**Table 3:** Shoulder lane traffic volume setting in the open shoulder scenario.

Shoulder width (m)	2.5	3	3.5
Traffic volume setting (VPH)	1200	1300	1600

### Indicators

The data were collected and output using the driving simulator (including lateral offset, speed, acceleration, and yaw velocity). For comparative analysis, the output data were processed using the ANOVA and Bonferroni multiple comparison methods in the SPSS software.

### Lateral offset

The lateral offset is the distance between the vehicle centroid and lane centerline. It is positive when the vehicle centroid is to the right of the centerline and negative when it is to the left. The greater the lateral offset, the more likely that vehicles in adjacent lanes will be disturbed while driving. Researchers can assess whether the inside shoulder width affects the lateral position of the vehicle and then recommend the optimal left hard shoulder width based on the lateral position by calculating the mean and standard deviation of the lateral position of all subjects on each highway.

### Speed

Based on the collected speed under different running conditions on the left hard shoulder, the mean value and standard deviation of the vehicle speed can be analyzed, and the stability and dispersion of the vehicle speed while driving can be explored. The narrower the speed dispersion, the higher the vehicle stability while driving. In this study, the speed performance of vehicles in the inside lane in both the presence and absence of traffic flow in the PTSU lane is considerably different and requires further investigation.

### Yaw velocity

**Fig 4:**
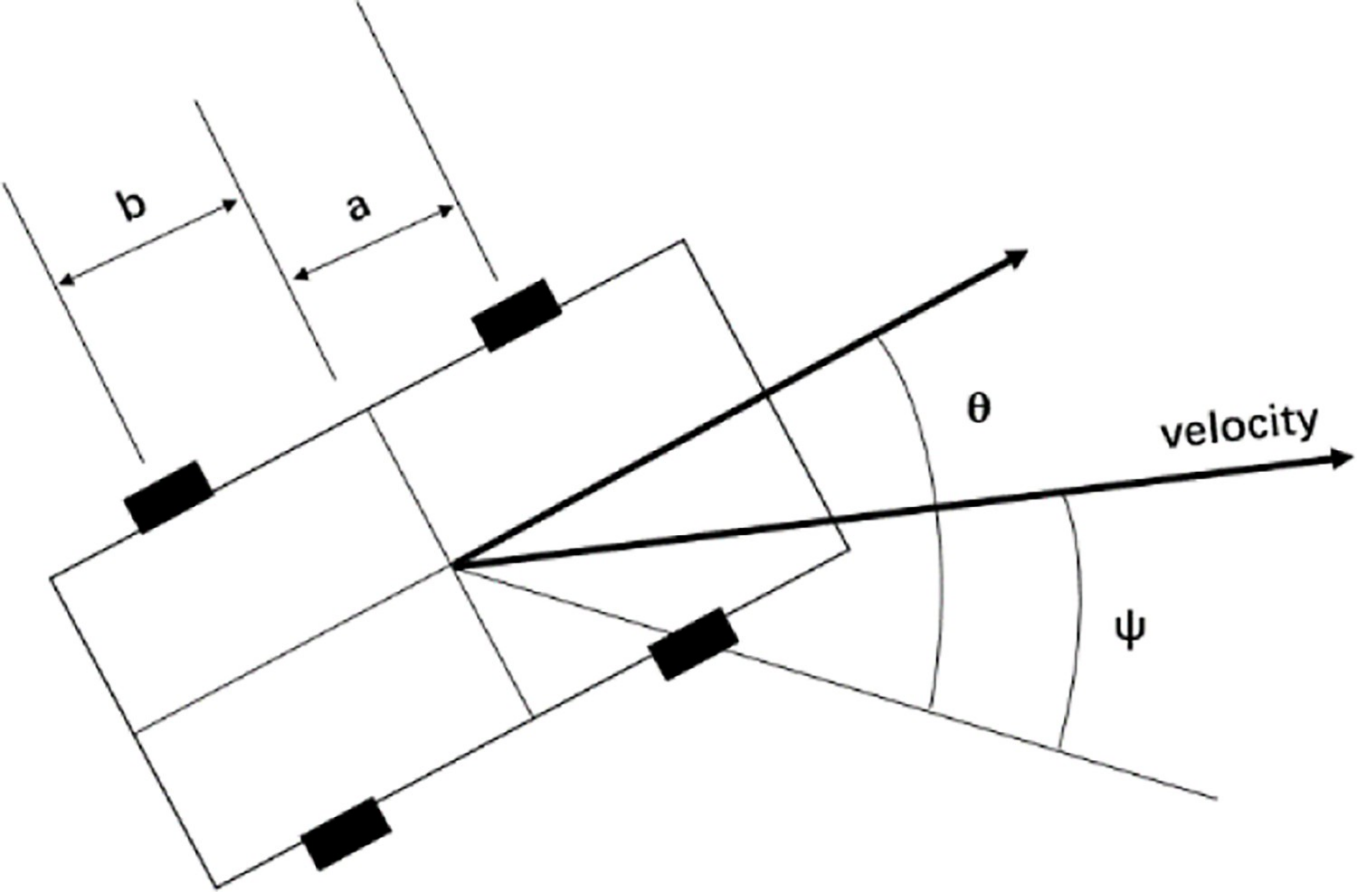
Dynamics of a road vehicle.

The yaw velocity is the rate of change in the vehicle rotation angle around the Z-axis perpendicular to the ground, and its magnitude represents the vehicle driving stability. The greater the vehicle yaw velocity while driving, the faster the change in the yaw angle, and the more prone the vehicle is to sideslip or tail flick. Fig 4 illustrates a four-wheel vehicle in which the front axle is located *a* m ahead of the center of gravity, and the rear axle is *b* m toward the rear from the center of gravity. The body of the car is facing in direction *θ*, while it is traveling in direction ψ. The contact area tire tread points in the direction of travel, but the wheel is aligned with the car, and the steering remains central. To compensate for this misalignment, the tires twist as they rotate, thereby generating lateral forces.

In the directional stability study, the angular velocity generated by the lateral force is the yaw velocity of the transverse pendulum, which is expressed as the angular velocity *ω* when the vehicle motion equation is

$$\frac{d\omega }{dt}=2k\frac{\left(a-b\right)}{I}\beta -2k\frac{\left(a^{2}+b^{2}\right)}{VI}\omega$$

$$\frac{d\beta }{dt}=-\frac{4k}{MV}\beta +(1-2k)\frac{\left(b-a\right)}{MV^{2}}\omega$$

where *ω* is the yaw velocity of the vehicle, *k* is the proportionality constant, *a* is the distance of the front axle of the vehicle from the center of gravity, *b* is the distance of the rear axle of the vehicle from the center of gravity, *V* is the vehicle speed, *I* is the yaw moment of inertia, *β* = *θ*−*ψ* is the vehicle overall angle, and *M* is the mass of the vehicle.

The requirement for vehicle driving stability is that both stiffness and damping are positive. This is always true if the center of gravity is ahead of the center of the wheelbase (*b* > *a*), and the vehicle remains stable at all speeds. The yaw velocity of the transverse pendulum influences the stability of the vehicle and has specific research significance.

### Procedures

In this study, considering the impact of the left hard shoulder width and vehicles traveling on the left hard shoulder as a temporary lane on the operation of vehicles in the adjacent inner lane of the highway, the subjects were asked to stay in the innermost lane of the highway. The adjacent lane of the left hard shoulder was chosen as the study target because the vehicles in this lane are the most affected by the left hard shoulder width and traffic flow during PTSU operation, and the fluctuation of the vehicle stability related indices is the most obvious.

According to the Highway Capacity Manual, the free-flow speed of a multilane highway is approximately 100 km/h [35]. The speed of the vehicles in the inner lane was higher than those in the other lanes. Therefore, the participants were asked to maintain a speed above 100 km/h to simulate the speed in the inner lane. A repeated measures design was used in the experiment, and each participant was instructed to drive at least four times on each roadway in both PTSU scenarios to improve data accuracy. The experiment was conducted in four rounds, where each participant would drive once on all roads in both scenarios in one round, and the roads driven in one round would not be repeated. The road driving sequence was disrupted to eliminate operational mistakes induced by regularity. The participants did not drive continuously for long periods during each round to ensure that they had sufficient energy during the experiment.

For data analysis, this study employed the one-way ANOVA to determine the significance of changes in the width of the left hard shoulder on the three variables (the confidence interval was set to 0.95). Three major assumptions were made for the ANOVA, namely, all samples were normally distributed, all had a common variance, and all were independent of one another [36]. However, numerous comparisons increase the rate of error. To control the error rate, SPSS was used to perform several post hoc tests for the ANOVA. The Bonferroni test is the most commonly used method for error control. After ANOVA, the Bonferroni multiple comparison test was used to assess the variability across groups of data to obtain the most diverse sets of data, examine the factors in each group, and remove incorrect hypotheses. The Bonferroni multiple comparison test was chosen because it allows repeated comparisons of groups of variables in the context of ANOVA, particularly in circumstances of uneven sample sizes or nonstandard linear comparisons, with suitable changes to allow the use of associated multiple comparisons. False-positive findings were eliminated by adjusting the threshold value. Owing to the large sample size and erroneous effect of differences in operation by different drivers on the results, the Bonferroni multiple comparison was required to correct the statistically incorrect judgments made by the ANOVA on the parameters as a whole to mitigate the error effect caused by the increased number of tests.

## Results

### Lateral offset

**Fig 5:**
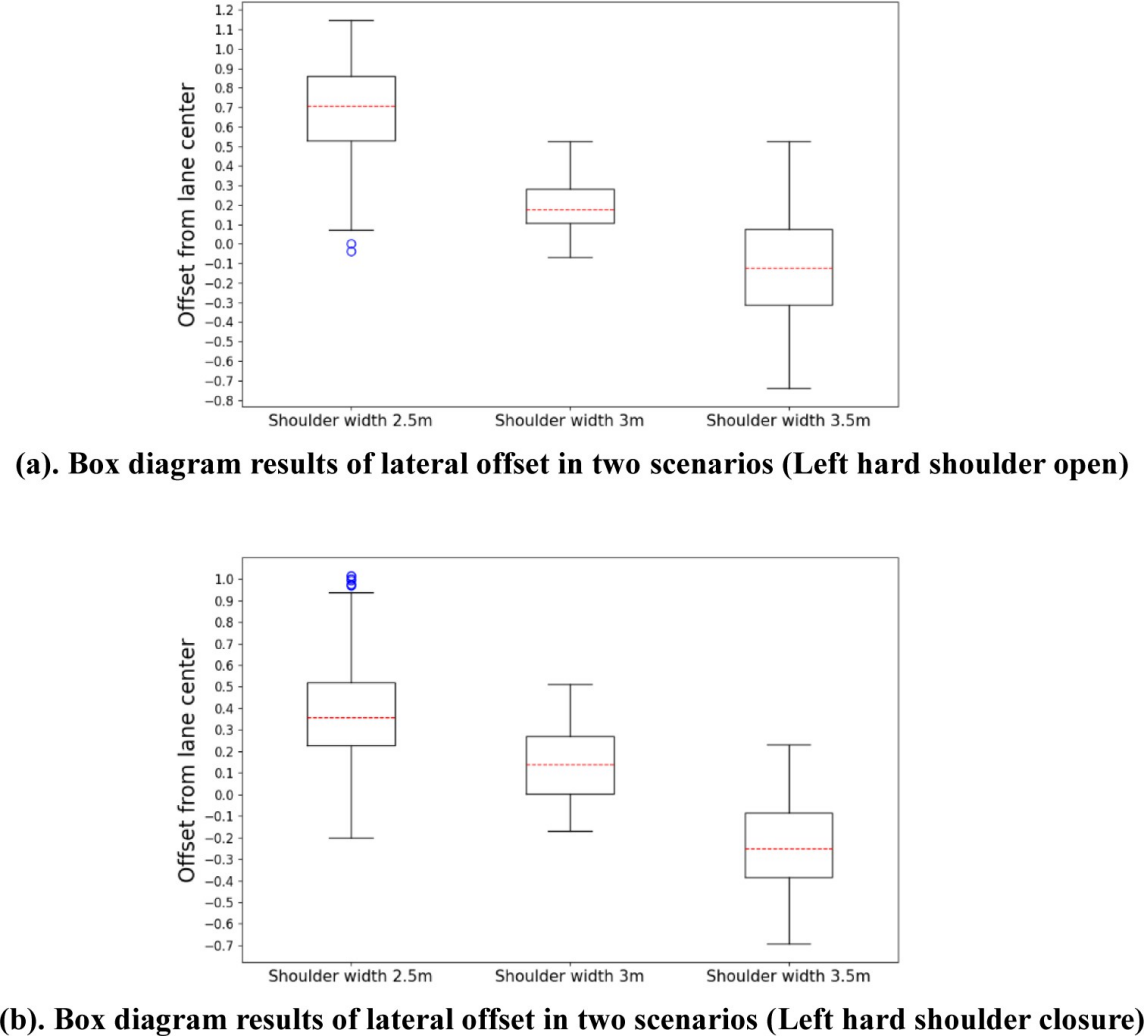
Box diagram results of the lateral offset in the two scenarios. (a) Box diagram results of lateral offset in two scenarios (Left hard shoulder open), (b) Box diagram results of lateral offset in two scenarios (Left hard shoulder closure).

Fig 5 displays the box plots of the lateral offset in the two scenarios. The results show that in open left hard shoulder scenario, the dispersion of the associated lateral offset data is maximal when the shoulder width is 3.5 m, and it is minimal when the shoulder width is 3 m. In contrast, in the closed left hard shoulder scenario, the dispersion difference between the three widths is not significant. In terms of the median, the vehicle median lateral offset is closest to 0 when the shoulder width is 3 m. This means that the vehicle travels closest to the centerline of the lane, but the vehicle position in the lane shifts to the left side of the lane as the left hard shoulder width increases in both the open and closed shoulder scenarios.

The significance of the effect of different left hard shoulder widths on the distance from the center of the lane for vehicles driving in the inside lane can be obtained through ANOVA comparison of the vehicle lateral offset output by the driving simulator, and the optimal left hard shoulder width can be determined.

The ANOVA results reveal that when the left hard shoulder is open, the model statistic (F (2956) = 804.486, p < 0.01) is significant, indicating that the width of the left hard shoulder has a substantial effect on the lateral offset. The Bonferroni post hoc multiple comparison test shows that the left hard shoulder widths of 2.5, 3, and 3.5 m between the lateral offsets differ considerably (p < 0.01). The shoulder width has a significant effect on the lateral offset when the left hard shoulder is closed (F (2956) = 815.373, p < 0.01). The Bonferroni post hoc comparison test exhibits significant differences in the lateral offset for all three groups (p < 0.01). The ANOVA results for the absolute value of the lateral offset show that the effect of the shoulder width is not significant (F (2956) = 278.869, p > 0.05). This indicates that when the left hard shoulder is closed, the distance from the centerline of the lane is less affected by the shoulder width, whether the vehicle is shifting to the left or right side of the lane.

The ANOVA findings exhibit significant differences between the datasets, and the mean value and standard deviation must be pooled for comparison to determine the optimal left hard shoulder width. The ANOVA results of the lateral offset are presented in Table 4, whereas Table 5 displays the mean values and standard deviations.

**Table 4:** ANOVA results of lateral offset.

ANOVA parameters					
	Quadratic sum	Degree of freedom	Mean square	F	Significance
Open shoulder (m)	101.820	2	50.910	804.486	1.310×10^−205^
Closed shoulder (m)	66.896	2	33.448	815.373	2.305×10^−207^

**Table 5:** Mean value (MV) and standard deviation (SD) results of lateral offset.

Scenarios	Results	Left hard shoulder width (m)		
		2.5	3	3.5
Open shoulder	MV (m)	0.682	0.307	-0.116
	SD (m)	0.232	0.253	0.269
Closed shoulder	MV (m)	0.402	0.143	-0.241
	SD (m)	0.236	0.164	0.201

The mean value of the lateral offset indicates that when the width of the left hard shoulder is 3 m, the lateral position of the vehicle is closest to the center of the lane, and the vehicle gradually shifts to the left side of the lane as the width of the shoulder increases. However, when left hard shoulder is open, the mean values of lateral offset corresponding to the three values of shoulder widths are smaller than those when the shoulder is closed, which means that vehicles prefer to drive on the left side of the lane. Through comparison, it was found that this may be because of the influence of traffic flow on the left hard shoulder. When there are vehicles on the left hard shoulder, drivers will choose to shift to the right side of the lane to maintain the perceived safety space on the left side. Whether the shoulder is open or closed, when the shoulder width is 3 m, the vehicles tend to move to the right side of the lane center. When the shoulder width is 2.5 m and 3.5 m, the lateral offset is larger, which means that the vehicle is farther away from the center of the lane than when the shoulder width is 3 m.

In both scenarios, the results of the lateral offset show that the standard deviation is the lowest when the left hard shoulder width is 3 m, indicating that the dispersion of the lateral offset is the lowest and the vehicle has the lowest left–right offset in the lane when the left hard shoulder is 3 m. The difference is that in the open left hard shoulder scenario, the standard deviation of the lateral offset is the largest when the shoulder width is 3.5 m, whereas in the closed left hard shoulder scenario, the standard deviation of the lateral offset is the largest when the shoulder width is 2.5 m. However, in any case, the lateral offset of the vehicle when the shoulder width is 3 m is the most ideal.

In summary, considering the mean and standard deviation of the lateral offset, the optimal width for the left hard shoulder should be 3 m.

### Speed

The speed data acquired in the experiments had little discrete variability; therefore, the conclusions were based mostly on the ANOVA and mean and standard deviation comparison analyses. The ANOVA results are presented in Tables 6 and 7 summarizes the mean value and standard deviation of the speed of driving on roads with varying widths on the left hard shoulder.

**Table 6:** ANOVA results of speed.

ANOVA parameters					
	Quadratic sum	Degree of freedom	Mean square	F	Significance
Open shoulder (m)	5072.687	2	2536.343	2546.205	1.620×10^−232^
Closed shoulder (m)	2815.667	2	1407.834	1060.299	2.297×10^−243^

**Table 7:** MV and SD results of speed.

Scenarios	Results	Left hard shoulder width (m)		
		2.5	3	3.5
Open shoulder	MV (m)	108.840	113.009	111.351
	SD (m)	1.235	0.649	1.427
Closed shoulder	MV (m)	105.832	111.410	109.317
	SD (m)	0.638	0.217	1.591

In the open left hard shoulder scenario, the ANOVA results reveal that the width of the left hard shoulder has a significant effect on the speed (F (2956) = 2546.205, p < 0.01). According to the Bonferroni post hoc comparison test results, all three sets of speed data are significantly different (p < 0.05). The results of the mean value indicate that the lowest and highest average speeds of the vehicle are achieved when the left hard shoulder widths are 2.5 m and 3 m, respectively. When the left hard shoulder width is 3 m, the standard deviation of the vehicle speed is the smallest (SD = 0.649), indicating that the dispersion of the vehicle speed is the smallest. Moreover, at 3 m, the overall speed of the vehicle is the highest and the speed stability is the best.

In the closed left hard shoulder scenario, the ANOVA results indicate that the width of the left hard shoulder has a significant effect on the vehicle speed when there are no vehicles on the left hard shoulder (F (2956) = 1060.299, p < 0.01). The Bonferroni post hoc comparison test results exhibit significant differences between the speeds in all three widths. The results of the mean value reveal that the average speed of the vehicle is the highest when the left hard shoulder width is 3 m and the lowest when it is 2.5 m. Furthermore, the standard deviation of the vehicle speed is the smallest when the left hard shoulder width is 3 m (SD = 0.217). Thus, the dispersion of the vehicle speed is the smallest when the left hard shoulder width is 3 m, and the overall state of the vehicle speed when driving in the lane is more stable than those at 2.5 m and 3.5 m. When the width of the left hard shoulder is 3.5 m, the data dispersion is the greatest and the overall vehicle speed is the most unstable. The difference between the open and closed left hard shoulder scenarios is that the standard deviation of the speed corresponding to each width is significantly lower in the closed scenario, indicating that the speed stability is better than that when the left hard shoulder is open.

Based on the results of the speed, the vehicle average speed is the fastest when the width of the left hard shoulder is 3 m. However, a higher average speed is not always beneficial, but the speed as a whole remains within the conventional interval, and a higher speed ensures a shorter traveling time to some extent. Moreover, the stability is the highest when the width of the left hard shoulder is 3 m, and the overall speed pattern is better than those of the other two.

### Yaw velocity

**Fig 6:**
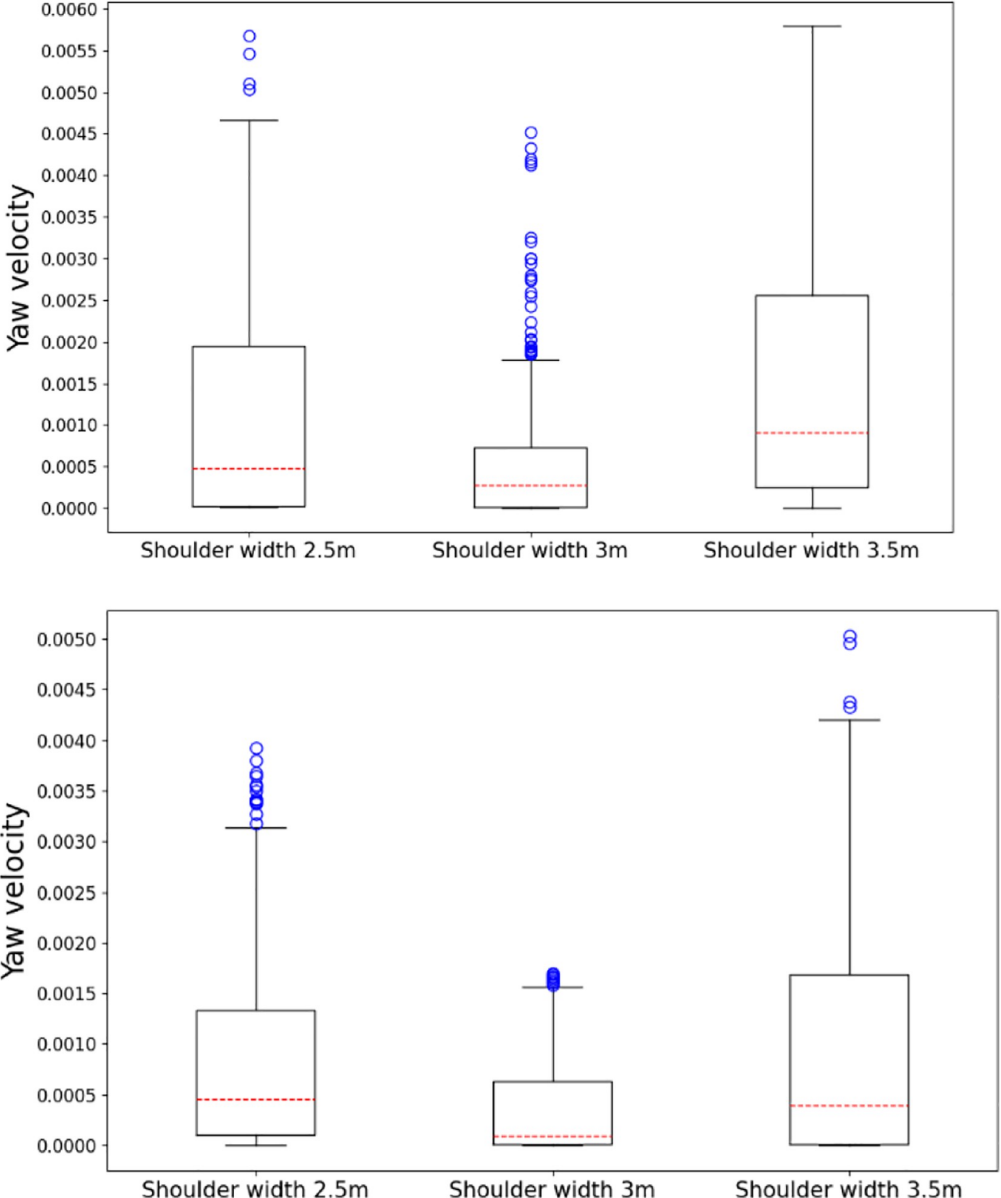
Box plot of yaw velocity in the two scenarios. (a) Box diagram results of yaw velocity in two scenarios (Left hard shoulder open), (b) Box diagram results of yaw velocity in two scenarios (Left hard shoulder closure).

Fig 6 displays a box plot of the yaw velocity for both cases with various shoulder widths. The results show that the dispersion of the yaw velocity data corresponding to the shoulder width of 3 m is the lowest, and the average value is the smallest in both scenarios. There are several outliers in the collected yaw velocity data. The degrees of freedom are minimal, the overall distribution is right skewed, and the median of the data is greater than the overall level. Consequently, the overall level of the data was based mainly on the mean value results.

By comparing the yaw velocity data extracted from the driving simulator, the effect of the left hard shoulder width on the vehicle driving stability can be determined. The ANOVA results are presented in Table 8, while the mean values and standard deviations are displayed in Table 9.

**Table 8:** ANOVA results of yaw velocity.

ANOVA parameters					
	Quadratic sum	Degree of freedom	Mean square	F	Significance
Open shoulder (m)	1.73×10^−4^	2	8.6×10^−5^	40.414	1.416×10^−17^
Closed shoulder (m)	4.18×10^−6^	2	1.971×10^−6^	1.195	0.303

**Table 9:** MV and SD results of yaw velocity.

Scenarios	Results	Left hard shoulder width (m)		
		2.5	3	3.5
Open shoulder	MV (m)	0.001423	0.000579	0.001527
	SD (m)	0.001799	0.000825	0.001578
Closed shoulder	MV (m)	0.001063	0.000912	0.001003
	SD (m)	0.001309	0.001141	0.001265

In the open left hard shoulder scenario, the ANOVA results show that the effect of the left hard shoulder width on the yaw velocity is significant (p < 0.01). The Bonferroni post hoc comparison test results indicate that the difference between the yaw velocities in the 2.5 m and 3.5 m widths is not significant (p > 0.05). However, the difference in the yaw velocity for the 3 m width is significant compared with the other two (p < 0.05). Therefore, it is necessary to further compare the mean value and standard deviation of the yaw velocity of the three different left hard shoulder widths to determine the differences so that the most appropriate width can be obtained. The mean value and standard deviation results reveal that the average yaw velocity is the lowest when the width of the left hard shoulder is 3 m. Moreover, the standard deviation of the yaw velocity is the smallest with the lowest data dispersion, indicating that the overall yaw velocity of the vehicle is the most stable when the left hard shoulder width is 3 m.

The results of the closed left hard shoulder scenario indicate that the mean value and standard deviation of the yaw velocity in the three shoulder widths are similar. This suggests that the shoulder width has no significant effect on the change in yaw velocity when the left hard shoulder is closed, and the ANOVA results obtained after repeated measurements support this conclusion (p > 0.05).

In both scenarios, the yaw velocity standard deviation of the left hard shoulder width of 2.5 m is similar to that at 3.5 m, which are all greater than that at 3 m. Therefore, it can be concluded that the stability of the yaw velocity of the vehicle in the inner lane is better when the width of the left hard shoulder is 3 m compared to 2.5 m and 3 m.

In summary, considering the results of the ANOVA, mean value, and standard deviation of the vehicle yaw velocity, the yaw velocity value is the most notable when the left hard shoulder width is 3 m.

## Discussion

Because of the precision of the experimental apparatus and limitations of the experimental procedure, the results may contain certain inaccuracies that merit additional discussion.

Previous studies have revealed the effects of different left hard shoulder widths on vehicle stability. According to previous research, the width of the left hard shoulder has a significant effect on the lateral offset of the vehicle [14]. Although the vehicle position does not change in a fixed direction as the shoulder width increases, it reciprocates to the left and right of the lane centerline [37]. Other studies have found that a narrow left hard shoulder reduces the vehicle speed; however, when the shoulder becomes considerably wide, the effect of the shoulder width on the speed is no longer apparent. This is because narrow shoulders physically bring objects such as roadside facilities closer to the lane, and the reduced lateral spacing of the edge of the travel lane extending to the nearest obstacle affects the speed choice of drivers in adjacent lanes [38].

All participants in the driving simulation experiment were requested to maintain their speed at over 100 km/h to replicate the speed environment in the inner lane of super multilane highways. The speed data analysis results in the closed left hard shoulder scenario indicate that the influence of the left hard shoulder width on the vehicle speed is significant, which is slightly different from the findings of Mecheri et al. [39]. This might be because the width of the left hard shoulder set by Mecheri et al. in their study was narrow (less than 0.75 m); thus, the effect of the change in the width of the shoulder was not significant. In contrast, the width set in this study is more than 2.5 m. When the width of the left hard shoulder exceeds a certain value, its effect on the speed becomes significant. In addition, this study supplemented the speed study of vehicles in the inside lane when the left hard shoulder is open. The results indicate that the effect of the left hard shoulder width on the speed is also significant, probably because vehicles driving on the left hard shoulder have a greater effect on the speed selection decisions of drivers in the inner lane.

The majority of the previous studies did not consider the influence of the width of the left hard shoulder on the vehicle yaw velocity. According to the study findings, the rate of change in the yaw velocity is not significant in the closed left hard shoulder scenario. One probable explanation is that the simulation scenario generated by the driving simulator is considerably different from that of the actual world, and the driving behavior is not significant enough [40]. This is because the safe environment and simple maneuverability provided by the driving simulator allowed participants to drive in a steadier condition than in reality, which resulted in a smaller yaw velocity data output by the experiment. The results of the post-experiment questionnaire survey supported this viewpoint as well. Among the 160 participants, 141 (88.13% of the total) said that it was simpler to operate the car on the driving simulator than in real life, and 127 (79.38% of the total) stated that it was less stressful to conduct actions such as overtaking and braking in the simulated situation than in real life. The yaw velocity should also be considered in engineering practice as one of the major indicators for assessing vehicle driving stability.

Physiological data such as heart rate and eye movement, which are commonly used in driving simulation experiments, were not gathered in this study because prior research has shown that the width of the left hard shoulder has no significant influence on the heart rate and eye movement of drivers in driving simulation experiments [19]. In the future, similar data may be acquired by instrumentation in the test of realistic scenarios to further investigate the effect of the left hard shoulder width on driver physiology.

The simulation scenario constructed in this study did not set sufficient supporting guidance facilities. In future research, we will focus on guidance facilities for further supplementation to dissect their effects on driver physiology and behavior under the PTSU strategy. In addition, this study only discussed the width range of the left hard shoulder. In the future, we will specifically investigate the geometric parameters of other PTSU facilities, including the upstream and downstream influence zones, entrance/exit transition zone, and ramp entrance/exit variable-speed lane. Furthermore, we will complement the effect of lane width on the setting of the left hard shoulder width.

This study mainly investigated the optimal width of the left hard shoulder of a ten-lane highway implementing PTSU. The experimental procedure and parameters were selected with reference to various regulations and specifications of a two-way ten-lane highway; therefore, the results of this study are limited to such road scenarios and may not be applicable to highways with different numbers of lanes or without PTSU. To fully evaluate the safety impact of the left hard shoulder width with PTSU, future research will consider different road conditions.

## Conclusions

In this study, by creating two simulated driving scenarios with the left hard shoulder open and closed and comparing the lateral offset, speed, and yaw velocity of the vehicle under various left hard shoulder widths, the optimal width of the left hard shoulder of a two-way ten-lane highway with PTSU was obtained. The main conclusions can be summarized as follows:

- When the left hard shoulder under PTSU is open, the width of the left hard shoulder has a significant effect on some specific driving stability parameters (lateral excursion, speed, and yaw velocity) of the vehicle, as evidenced by the one-way ANOVA results, Bonferroni post hoc test results, and mean and standard deviation comparison results.
- When the left hard shoulder under PTSU is closed, the influence of the shoulder width on the yaw velocity is insignificant. Compared to the data obtained when the left hard shoulder is open, the variation in the yaw velocity is mostly influenced by the traffic flow on the left side hard shoulder. The yaw velocity is optimal when the shoulder width is 3 m, which may be because the 3 m width can provide the driver of the inner lane with sufficient left side safety perception distance; however, as the shoulder width increases, the vehicle speed increases, whereas the vehicle maneuverability decreases and the yaw velocity increases.
- For the specific width parameter setting of the hard shoulder on the left side of a super multilane highway where PTSU is implemented, the study concluded that the optimal width of the left hard shoulder should be 3 m.

The study findings provide some theoretical justification for changing the present design specifications of the super multilane PTSU shoulder width in places where cars drive on the right; however, more research needs to be conducted in the future. We need to investigate the connection between the stability of cars traveling in PTSU lanes and the set shoulder width, as well as the appropriate left hard shoulder width based on traffic capacity and highway design speed.

## Supporting information

S1 Appendix (PDF)
