# Supplementary material for: Research on left hard shoulder width of super multilane highway based on PTSU operation
Source: PLoS One. 2023 Jun 23;18(6):e0287606. doi: 10.1371/journal.pone.0287606 (PMC10289350; doi:10.1371/journal.pone.0287606)
Supplement: S1 Appendix — (PDF) [file pone.0287606.s001.pdf]

《基于 PTSU 策略的超多车道高速公路左侧硬路肩宽度研究》实验受试者知情同意书

尊敬的实验参与者：

您好，

作为实验的参与者，您有权了解有关本实验的信息，以帮助您决定是否愿意参加本实验。请您认真阅读，如有疑问可以向实验室管理员咨询。

实验目的：分析驾驶人在实施 PTSU 策略的超多车道高速公路上的行驶稳定性。

实验对象：男性、已成年、身体健康、持有 C1 驾照、驾龄在 2 年以上。

实验过程：本实验为驾驶模拟实验。在实验开始前，您需要在实验室人员的帮助下熟悉驾驶模拟仪的操作。在实验开始后，您只需在道路场景中按习惯保持正常驾驶即可，您预计会参与 4 轮及以上的实验，在每轮实验结束后，您会得到充分的休息时间。如果在实验中出现任何不适感，您随时可以向实验人员提出终止实验。在实验完成后，您会拿到一份关于实验的问卷调查，麻烦您认真填写。

实验数据：实验中涉及您个人隐私的数据我们会确保做好保密工作，其中关于年龄、驾龄、驾驶模拟结果等数据可能会作为论文的必要数据出示。但请您放心，此类数据经过处理和分析，仅会作为无个人标识的数据集出示。若有任何疑问，您可以通过电话 13951035187 和实验室管理员马健霄教授联系。

感谢您的配合！

受试者知情同意声明：

我已仔细阅读了本知情同意书，了解实验的各项内容和细节。在仔细阅读上述内容后，经过充分的考虑，我自愿成为本研究的受试者，配合实验人员参与本场实验。

受试者签名：

联系电话：

日期：

实验员签名：
